# Cardiac arrest in a toddler treated with propranolol for infantile Hemangioma: a case report

**DOI:** 10.1186/s13052-017-0421-5

**Published:** 2017-11-17

**Authors:** Alvise Tosoni, Mario Cutrone, Maurizio Dalle Carbonare, Andrea Pettenazzo, Giorgio Perilongo, Stefano Sartori

**Affiliations:** 1 Department of Women’s and Children’s Health, Pediatric Intensive Care Unit, University Hospital of Padua, via Giustiniani 3, 35128 Padova (PD), Italy; 2 Pediatric Dermatology Unit, “Dell’Angelo” Hospital, Venice, Italy; 3 Research&Innovation s.r.l., Padua, Italy; 4 Department of Women’s and Children’s Health, Pediatric Neurology and Neurophysiology, University Hospital of Padua, Padua, Italy

**Keywords:** Adrenergic beta-blockers, Asystole, Child

## Abstract

**Background:**

Propranolol has become the first-line treatment for complicated Infantile Hemangioma (IH), showing so far a good risk-benefit profile.

**Case presentation:**

We report the case of a toddler, on propranolol, who suffered cardiac arrest during an acute viral infection. She had a neurally-mediated syncope that progressed to asystole, probably because of concurrent factors as dehydration, beta-blocking and probably individual susceptibility to vaso-vagal phenomena. In fact a significant history of breath-holding spells was consistent with vagal hyperactivity.

**Conclusions:**

The number of patients treated with propranolol for IHs will increase and sharing experience will help to better define the safety profile of this drug.

## Background

Infantile hemangiomas (IH) are the most common vascular tumors of childhood affecting about 5% of infants. They are benign tumors that usually appear in the first few weeks of life and they go through a rapid proliferative phase that lasts several months before a slower involution phase begins. Many of these lesions resolve spontaneously with few residuals. Nevertheless some IHs can be functionally significant for size and/or location and therefore deserve medical treatment. Since propranolol has been discovered to clearly reduce hemangiomas it has gained a primary role in the treatment of high-risk IHs and so far it has shown a favorable risk-benefit profile [1].

A pediatric propranolol formulation (Hemangeol/Hemangiol) has been approved by FDA (Food and Drug Administration) and EMA (European Medicine Agency) in 2014 specifically for the treatment of IH.

We report the case of a toddler who suffered a cardiac arrest while on treatment with propranolol for IH.

## Case description

**Fig. 1:**
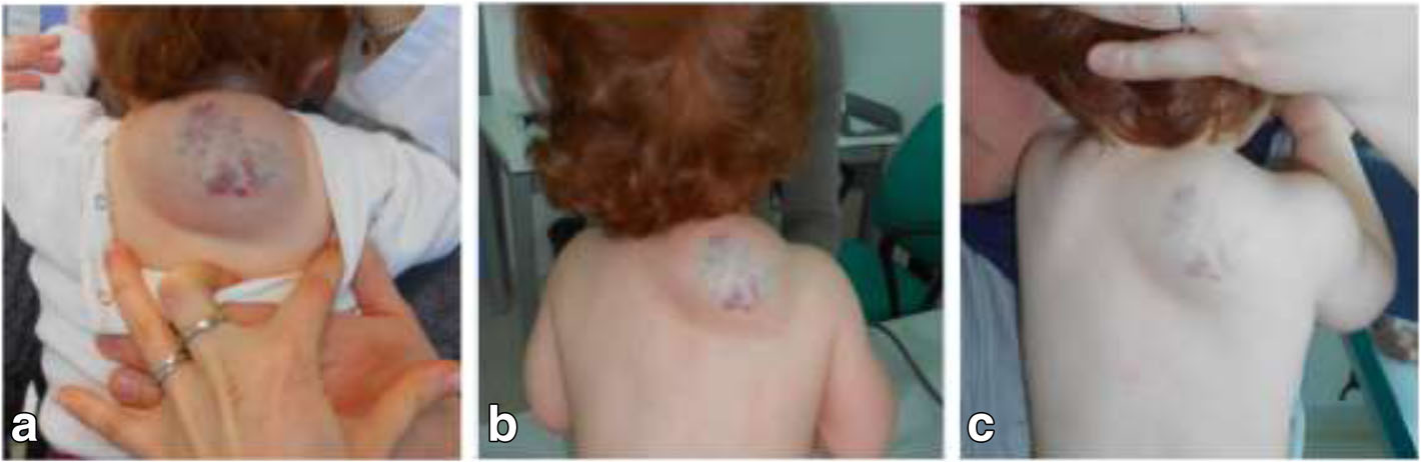
**a** before starting medical treatment with propranolol. **b** 8-day follow-up. **c** 37-day follow-up

A 14-month-old white female toddler was referred to a service of pediatric dermatology for a wide IH on the interscapular back area. The lesion appeared after birth and grew rapidly in the first few months. She was first followed in another center where laser therapy was attempted unsuccessfully. On examination the hemangioma was described as remarkable in size, tense and not showing clear signs of involution yet. For its position the lesion was uncomfortable and exposed to possible trauma as well as pressure and rubbing when the child was laying on the back (Fig. 1a). Even if she was not an infant anymore, she was deemed to meet the criteria for medical treatment in order to avoid possible complications and a spontaneous involution with potentially aesthetical damages. A decision was taken to try oral beta-blocker.

History and physical examination were unremarkable for cardiac problems; anyway she underwent an ECG and an echocardiogram, which were normal. Treatment was initiated at a dose of 0.5 mg/kg/die (in 2 daily doses) and increased to a target dose of 2 mg/kg/die during a two-day hospital admission in order to monitor for possible side effects (in particular bradychardia, hypotension) as recommended by a group of experts [2]. The drug was well tolerated, so she was discharged home. Propranolol was prepared as a solution (1 mg/ml) by a pharmacist according to medical prescription. Parents were informed to hold therapy in case of gastrointestinal infections or any other case of reduced oral intake to avoid the risk of hypoglycemia. The response to treatment was significant as documented at two follow-up visits at 8 and 37 days from initiation (Fig. 1b-c).

Forty days into the treatment the child developed an upper respiratory tract infection. After a couple of days she was reported to have some fatigability, lack of appetite and decreased oral fluid intake. The parents were planning to withhold the evening dose of propranolol, but 8 h after the morning dose, the child fainted without regaining consciousness, during an effort to pass stool. EMS (Emergency Medical Services) was called and chest compressions were started in few minutes by a trained neighbor. On arrival EMS found her asystolic and carried on cardiopulmonary resuscitation for 20 min before return of spontaneous circulation. She arrived to pediatric ICU hemodynamically stable without requirement of inotropic support. Blood work showed hypernatremia (Na 156 mmol/L), other electrolytes and glycaemia were in normal ranges. Hemoglobin was normal (121 g/L, MCV 78.4 fL). White blood cell count was unremarkable (7700/uL) and CRP only mildly elevated (16 mg/L). Adenovirus and Rhinovirus were isolated from nasal swab. Parents provided us with the bottle of propranolol solution whose concentration was tested and confirmed to be correct. Twelve hours after admission she developed status epilepticus despite all neuroprotective strategies were taken. A brain MRI revealed severe diffuse hypoxic-ischemic injuries. ECGs, ECG-Holter, echocardiogram were confirmed to be normal. She tested negative for known mutations in a panel of 86 genes causing cardiomyopathies and channelopathies implicated in arrhythmias (Table 1). She also was tested for genetic polymorphisms possibly responsible for a slower propranolol metabolism (Table 2). She was found to carry four single nucleotide polymorphisms (SNP) in genes encoding for enzymes involved in phase I (CYP2D6, CYP2C19) and II (UGT1A7, UGT2B7) metabolism, and one SNP in a gene (ORM1) encoding for a plasma protein (alpha-1-lacid glycoprotein 1) involved in the binding and transport of many drugs.

**Table 1:** Panel of genes responsible for cardiomyopathies and channelopathies implicated in arrhythmias. Mutation of SCN9A has been associated to “Paroxysmal Extreme Pain disorder” which can present as breath-holding spells and episodes of extreme bradycardia in infancy

Arrhythmias	Cardiomyopathies
ABCC9	ACTC1
AKAP9	ACTN2
ANK2	ANKRD1
CACNA1C	BAG3
CACNB2	BRAF
CALM1	CALR3
CASQ2	CAV3
CAV3	CRYAB
DSC2	CSRP3
DSG2	DES
DSP	DMD
GJA5	DTNA
GPD1L	EYA4
HCN4	FKTN
JUP	GATAD1
KCNA5	GLA
KCND3	JPH2
KCNE1	KRAS
KCNE2	LAMA4
KCNE3	LAMP2
KCNH2	LDB3
KCNJ2	MIB1
KCNJ5	MYBPC3
KCNQ1	MYH6
KCNQ1	MYH7
LMNA	MYL2
NPPA	MYL3
NUP155	MYOZ2
PKP2	MYPN
RYR2	NEXN
SCN1B	NF1
SCN2B	NNT
SCN3B	NRAS
SCN4B	PLN
SCN5A	PRDM16
SNT1	PRKAG2
TGFB3	PTPN11
TMEM43	RAF1
TRDN	RBM20
SCN9A	RIT1
	SGCD
	SOS1
	TAZ
	TCAP
	TMPO
	TNNC1

**Table 2:** Single nucleotide polymorphisms (dbSNP) identified in genes encoding enzymes involved in propranolol’s metabolism and pharmacokinetics

Gene	locus	dbSNP
CYP2D6	22q13.1	rs16947
CYP2C19	10q24	rs17878459
ORM1	9q32	rs1126801
UGT1A7	2q37	rs374860015
UGT2B7	4q13	rs138733571

Notably, a history of frequent pale and cyanotic breath-holding spells (BHS) emerged.

The patient was transferred out of the ICU on day 15 after admission. She was finally discharged from the hospital 55 days after the event with poor neurologic outcomes as spastic tetraparesis, epilepsy and severe cognitive impairment.

## Discussion

We report the case of a 14-month old toddler on treatment with oral propranolol for IH who, during a viral illness, suffered a cardiac arrest, which resulted in a severe neurologic outcome. To our knowledge there is only another report of a case of cardiac arrest in a child with bronchiolitis while on propranolol for IH [3].

A recently published RCT [4] has confirmed that oral propranolol at a dose of 3 mg/kg/day for 6 months is effective. In this study only one patient on propranolol (1/401) was described to suffer a serious adverse event like bradycardia in the context of enterocolitis. To follow, the American Academy of Pediatrics published a comprehensive guidance for the diagnosis and management of IHs [5]. The authors report how propranolol has become the first-line drug for complicated IHs. A complete history and physical examination, focusing especially on cardiac and respiratory problems, is necessary to investigate the child’s suitability for treatment. Even though many clinicians prefer to have an ECG or even a cardiology consultation, a cardiac screening would appear of limited value in face of unremarkable cardiac history and examination. Reported contraindications are sinus bradycardia, hypotension, heart-block, heart failure, cardiogenic shock, reactive airways, hypoglycemia, hypersensitivity to the drug. Most commonly reported side effects are diarrhea, sleep disorders, cool extremities, reactive airways, hypoglycemia. Predictable important risks as bronchospasm, bradycardia, hypotension and hypoglycemia are infrequent and rarely lead to treatment discontinuation. It is important to note that, as at now, there are not precise guidelines on how to initiate the treatment and how to monitor for side effects. Yet, in recent publications, authors have suggested to start at 0.5-1 mg/kg/day and to slower and cautiously increase by 0.5-1 mg/kg/day to a target dose of 2-3 mg/kg/day over few weeks. Heart rate and blood pressure should be monitored in the first 2-3 h after the first intake and at each dose increase. Suggestions to initiate the treatment as inpatients are made only for infants younger than 2 months, weight less than 2000 g, infant with inadequate social support, or comorbidities [6, 7]. 

Here we report the case of a severe cardiac adverse event in an otherwise healthy child on treatment with propranolol for IH. We believe the cardiac arrest was the consequence of an unfortunate combination of factors. The patient suffered from breath-holding spells and despite being usually a benign condition, it is potentially associated with neurally-mediated (vasovagal) phenomena [8]. In fact a painful or distressing event may induce a vagally mediated cardiac inhibition with prolonged asystole usually terminated by escape junctional beats or recovery of sinus rhythm. There are even reported cases of successful treatment of complicated severe BHS with pacemaker [8–10]. The patient performed a Valsalva’s maneuver that triggered a neurally-mediated syncope which probably she didn’t recover from because she was dehydrated and beta-blocked so that it progressed to asystolic cardiac arrest. Finally the patient could have been a slower metabolizer of the drug, maintaining therefore higher plasmatic concentration than expected. Her CYP2D6’s variant is associated with a phenotype with decreased enzymatic activity [11–13], but we don’t know what is the effect of the other SNPs we identified and we can only speculate they could affect the pharmacokinetics of propranolol. Yet it is important to note that she didn’t show the signs of beta-blocker intoxication (i.e. hypotension, bradycardia, ECG abnormalities, hypoglycemia) once spontaneous circulation was restored.

## Conclusions

The number of patients treated with systemic beta-blockers for IHs is expected to grow and some clinicians might even consider offering this effective treatment to children beyond the proliferation phase. In fact off-label use of propranolol after 5 months of age (as for our case) has frequently been described as successful in the literature [14–17]. The experience with this drug is therefore accumulating and we will gain more and more information on its effectiveness [18] and its possible side effects. If more of such severe adverse events were to be reported, we believe stricter indications and contraindications should be discussed.

Meanwhile if the treatment is to be offered to a child older than 6 months, a history of breath-holding spells needs to be investigated. We believe that this group of children should be treated with great caution since they have a higher risk to develop vagally mediated bradycardia.

It could be worth discussing if monitoring should be extended longer than few hours after initiation and if a Holter-ECG is to be considered once the target dose has been reached in order to detect possible episodic bradycardia, which might prompt dose reduction or treatment’s discontinuation.

As already recommended, it is important to strongly advise parents to seek for a doctor’s opinion and follow-up in case of intercurrent infections while on treatment; propranolol therapy should be temporarily withheld in cases of poor oral feeding, diarrhea and bronchitis/bronchiolitis. [5, 6]

## Abbreviations

BHS: Breath holding spells
ECG: Electrocardiogram
ICU: Intensive Care Unit
IH: Infantile Hemangioma
SNP: Single nucleotide polymorphism
